# Effectiveness of ombitasvir/paritaprevir/ritonavir, dasabuvir for HCV in HIV/HCV coinfected subjects: a comprehensive analysis

**DOI:** 10.1186/s12985-018-1114-4

**Published:** 2019-01-17

**Authors:** Jingjing Wu, Peng Huang, Haozhi Fan, Ting Tian, Xueshan Xia, Zuqiang Fu, Yan Wang, Xiangyu Ye, Ming Yue, Yun Zhang

**Affiliations:** 1 Department of Epidemiology and Biostatistics, School of Public Health, Nanjing Medical University, Nanjing, 211166 China; 2 Key Laboratory of Infectious Diseases, School of Public Health, Nanjing Medical University, Nanjing, 211166 China; 3 Institute of Epidemiology and Microbiology, Huadong Research Institute for Medicine and Biotechnics, Nanjing, 210002 China; 4 Faculty of Life Science and Technology, Kunming University of Science and Technology, Yunnan, 650550 China; 5 Department of Infectious Diseases, The First Affiliated Hospital of Nanjing Medical University, Nanjing, 210029 China

**Keywords:** 3DAA, HCV, HIV, SVR12

## Abstract

**Background:**

Data on the treatment of patients with hepatitis C virus (HCV)/human immunodeficiency virus (HIV) coinfection remains limited. A comprehensive analysis was performed to evaluate the efficacy and safety of ombitasvir (OBV)/paritaprevir (PTV)/ritonavir(r) ± dasabuvir (DSV) ± ribavirin (RBV) for treatment in HCV/HIV coinfected patients.

**Methods:**

We systematically searched and included studies that enrolled patients with HIV/HCV coinfection using the OBV/PTV/r ± DSV ± RBV regimens and reported sustained virological response after 12 weeks (SVR12) end-of-treatment. Heterogeneity of results was assessed and pooled SVR rates were computed with 95% confidence intervals (95%CI). Subgroup analysis and assessment of publication bias through Egger’s test were further performed.

**Results:**

Ten studies containing 1358 coinfected patients were included in this study. The pooled estimate of SVR12 was 96.3% (95%CI: 95.1–97.4). Subgroup analysis showed that pooled SVR12 rate was 96.2% (95% CI: 94.8–97.4) for patients with genotype (GT) 1 and 98.8% (95% CI: 95.1–100.0) for those with GT4. The SVR12 rates for the treatment-naïve (TN) and treatment-experienced (TE) patients were 96.8% (95% CI, 94.8–98.5) and 98.9% (95% CI, 96.4–100.0), respectively. Pooled SVR12 rate was 97.8(95%CI: 94.6–99.8) for patients with cirrhosis and 96.7% (95%CI: 95.3–97.8) without cirrhosis. The pooled incidence of any adverse events (AEs) and serious adverse events (SAEs) was 73.9% (95%CI: 38.1–97.6) and 2.7% (95%CI: 0.0–9.5). Publication bias did not exist in this study.

**Conclusions:**

The comprehensive analysis showed high efficacy for the OBV/PTV/r ± DSV ± RBV regimen in patients coinfected with HIV and HCV, regardless of genotypes, history of treatment and the presence or absence of cirrhosis.

**Electronic supplementary material:**

The online version of this article (10.1186/s12985-018-1114-4) contains supplementary material, which is available to authorized users.

## Background

Worldwide, an estimated 5–10 million individuals with human immunodeficiency virus (HIV) are coinfected with hepatitis C virus (HCV) [1]. HIV coinfection can accelerate the progression of hepatitis C to cirrhosis, hepatocellular carcinoma (HCC) and liver failure [2]. For more than a decade, treatment of HCV with pegylated interferon (peg IFN) plus ribavirin (RBV) has been recommended for patients coinfected with HIV, but a poor rate of sustained virological response (SVR) has been achieved (17–36%). In addition, treatment-limiting adverse effects, and lots of contraindications have limited broad availability of HCV treatment in patients with coinfection [3, 4]. The introduction of direct-acting antiviral agents (DAAs) is a breakthrough in the treatment of HCV infection [5]. This therapeutic advance provided new opportunities for the treatment of patients coinfected by HCV and HIV [6].

Recently, the emergence of second-generation DAAs changed the paradigm of hepatitis C treatment with SVR rates exceeding 90%, and regardless of the presence of cirrhosis, former non-response [7, 8]. In contrast to first generation DAAs, these agents have added polymerase and NS5A inhibitors and can be used in combination, which increases their potency and ability to overcome the genetic barrier for resistant strains [9, 10]. The 3-DAA regimen of ombitasvir(OBV)/paritaprevir(PTV)/ritonavir(r) ± dasabuvir (DSV) ± ribavirin(RBV) was approved for therapy of both treatment-naive (TN) and treatment-experienced (TE) patients infected with HCV genotype (GT) 1 and 4 by the end of 2014 [11].

The interferon-free, all-oral regimen of the 3-DAA have achieved response rates from 92 to 100% in patients monoinfected with HCV GT1, including those with historically difficult-to-cure hosts and disease characteristics such as prior peg IFN-plus-RBV treatment failure, IL28B non-CC genotype, and cirrhosis [12, 13]. Among HIV/HCV coinfected patients, the 3-DAA regimen of OBV/PTV/r + DSV demonstrated 97% SVR rates in GT 1a-infected patients treated with a 12-week or 24-week regimen with or without RBV and 100% in GT1b-infected patients treated for 12 weeks without RBV [14].

The all-oral two direct-acting antivirals (2-DAA) of OBV/PTV/r + RBV have achieved response rates from 94 to 97% in patients monoinfected with HCV GT4 with or without compensated cirrhosis [15, 16]. Among HIV/HCV GT4 coinfected patients, the regimen of OBV/PTV/r + RBV achieved SVR rates up to 94.7 and 100% in 12 weeks of treatment for patients without cirrhosis and with compensated cirrhosis [6].

However, there is currently no systematic study to evaluate the clinical characteristics of OBV/PTV/r ± DSV ± RBV regimens. Our post hoc analysis is aimed to assess the efficacy and safety of OBV/PTV/r ± DSV ± RBV in an expanded study whose population consisted of 1358 patients with HIV/HCV genotype 1 or genotype 4 coinfection.

## Methods

### Literature search

A systematic search in PubMed, Web of Science and the Cochrane Library was conducted to identify relative studies. We searched with MeSH terms and keywords for published articles. No filters regarding languages or publication date were used. The search strategy includes the following terms: “hepatitis C” (e.g. “HCV”; “hepatitis C virus”; “hepacivir*”; “hepatitis C virus infection”); “human immunodeficiency virus” (e.g.“HIV”;“AIDS”); and “ombitasvir/ paritaprevir/ ritonavir +/- dasabuvir +/- RBV”. The manual search was also performed by checking the references of included studies and published narrative reviews for potentially eligible studies.

### Inclusion and eligibility criteria

We collected studies assessing the efficacy and safety of the DAAs in treating HCV and HIV coinfected patients. The studies would be included if they met all the following criteria: (i) study subjects were patients infected with both HCV and HIV; (ii) interventions were the combined DAAs regimen OBV/PTV/r ± DSV ± RBV without pegylated interferon; (iii) the study reported the efficacy and safety outcomes; and (iv) patients coinfected with HCV/HIV should be treated with antiretroviral therapy (ART). Studies would be excluded if they met anyone of the following items: (i) participants were coinfected with other viruses (e.g. HBV) or had advanced diseases(e.g. liver or kidney transplantation); (ii) DAAs was received as mono-therapy drug or in combination with other protease inhibitors; (iii) The sample size is less than 10; (iv) pharmacokinetics or pharmacodynamics studies; (v) the studies were published in a non-English journal; or (vi) conference abstracts without full text.

### Study selection

Two reviewers independently reviewed titles and abstracts for choosing the studies. Full articles were ascertained if the decision could not be made based on titles and abstracts. Disagreement between the two reviewers was determined by consensus with a third party.

### Data extraction

Data were extracted by two investigators to maintain uniformity. Relevant data included the first author’s name, year of publication, study design, treatment regimen, number of patients, HCV GT, duration of treatment, history of treatments, presence of cirrhosis, age, sex, HCV RNA levels, mean Baseline CD4+ T-cell counts, SVRs at weeks 12 (SVR12), viral relapse, viral breakthrough and safety outcomes included adverse events (AEs) and serious adverse events (SAEs).

### Statistical analysis

The proportions of SVR12 rates, viral relapse, viral breakthrough and safety outcomes were pooled using the Wilson score method. These rates were calculated with a 95% confidence interval (CI) in a fixed effect model. Heterogeneity across the included studies was assessed using the Cochran Q-statistics and I^2^ statistics, if the result of *P* < 0.10 or *I*^*2*^ > 50%, random effects model was employed. To effectively evaluate the efficacy and safety of DAA regimen, we conducted subgroup analyses of SVR12 by different duration of treatment, regimens, genotype of HCV, history of treatments, and the presence or absence of cirrhosis. Publication bias was assessed using egger test and funnel plot. The threshold for statistical significance was set at *P*-value< 0.05. All statistical analyses were conducted using the meta package in R (3.5.1).

## Result

### Search results and study characteristics

**Fig. 1:**
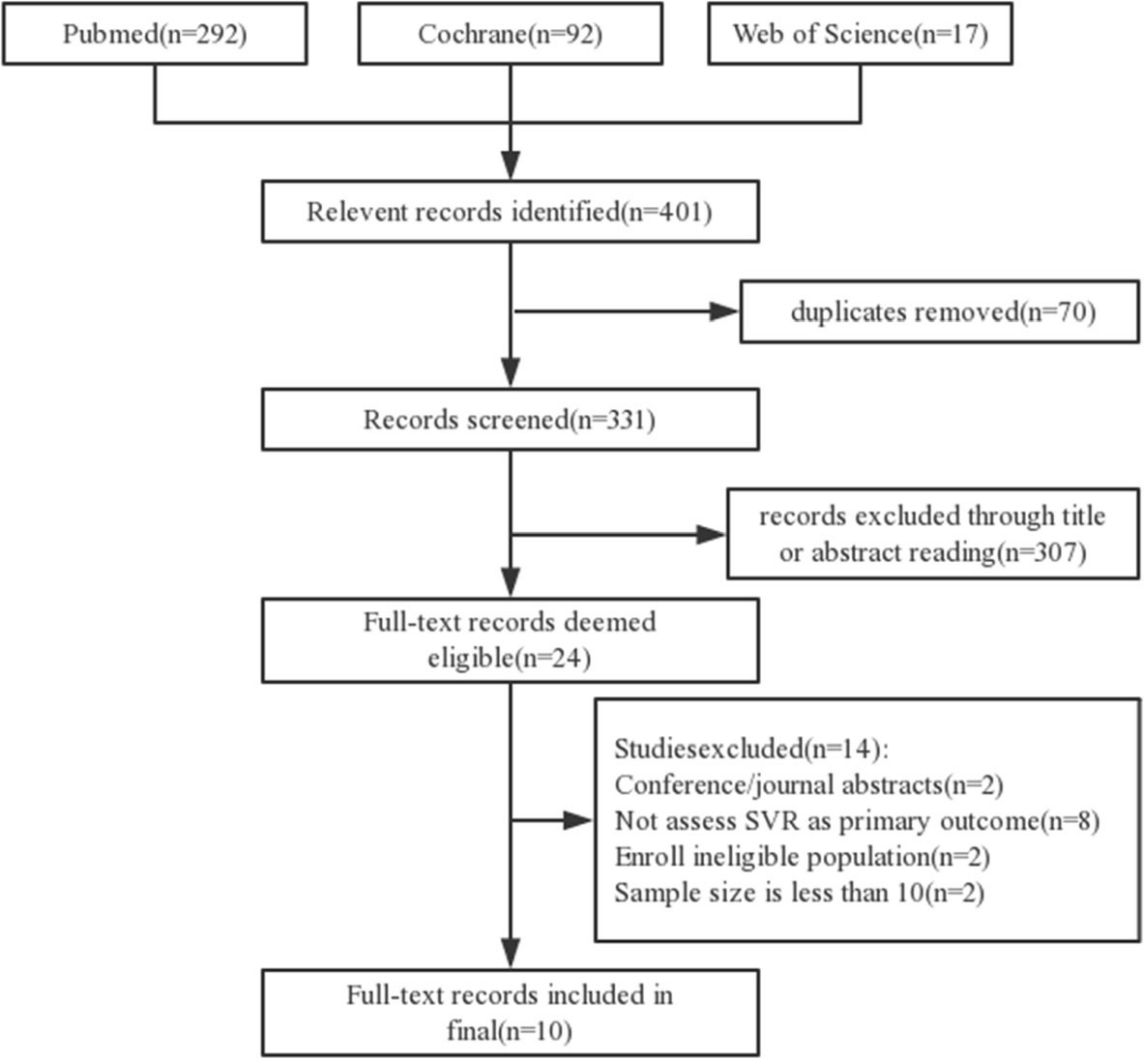
PRISMA flow chart of the literature search and selection methods used. *SVR: sustained virological response

Our electronic search retrieved 401 records. After removing duplicate studies, the titles and abstracts of the remaining articles were screened. A total of 24 articles were selected for full-text reading. According to the inclusion and exclusion criteria, 14 studies were excluded: 2 were conference/journal abstracts; 8 did not assess SVR as primary outcome; 2 enrolled ineligible population; 2 were only one or two subjects. Manual search did not add more eligible studies. After two phases of screening, ten studies [2, 6, 17–24] were added to this study (Fig. 1).

In total, included studies represented data from eleven countries and regions: seven from Europe (France, Germany, Italy, Russia, Spain, the United Kingdom and Vienna), three from North America (2 United States and 1 Canada) and one from New Zealand. For studies having the information of genotype of HCV, history of treatments, and the presence or absence of cirrhosis, we found that overall 1076 patients were infected with HCV genotype1 and 156 patients were infected with HCV genotypes 4; 230 patients were cirrhosis, and 935 patients were non-cirrhosis; 440 patients were TN, and 285 patients were TE. Data on study characteristics were shown in Table 1.

**Table 1:** Main characteristics of the studies and patients enrolled in this comprehensive analysis

Study	Study design	Study period	Regimen	N	SVR12 (%)	Mean ± SD (Age, year)	Male (%)	Cirrhosis (Yes|No)	TN|TE	GT1|GT4
Rios (2018)	observational study	2014.12–2016.8	OPr ± D ± R	440	420 (95.5)	51.0 ± 1.2	NA	101|339	NA	299|125
Pineda (2018)	cohort	2015.3–2017.1	OPrD±R	182	172 (94.5)	51.0 ± 1.2	151 (82.9)	69|113	101|81	GT1
Massimo (2017)	cohort	NA	OPrD±R	210	203 (96.7)	53.0 ± 7.8	157 (74.8)	23|187	96|114	GT1
Rockstroh (2017)	clinical trial	2015.7–2015.12	OPrD±R	228	221 (96.9)	50.0 ± 7.2	171 (75.0)	23|205	151|76	200|28
Bhattacharya (2017)	observational cohort	2016.4	OPrD±R	89	79 (88.8)	61.4 ± 6.1	89 (100)	14|75	79|10	GT1
Sulkowski (2015)	clinical trial	2013.9–2014.8	OPrD+R	63	58 (92.1)	50.9 ± 7.2	58 (92.0)	NA	42|21	GT1
Montes (2017)	observational study	2015.4–2015.12	OPr ± D ± R	88	87 (98.9)	51.4 ± 1.0	67 (76.1)	NA	NA	NA
Wyles (2017)	clinical trial	2014.12–2015.7	OPrD+R	22	22 (100.0)	NA	17 (77.3)	3|19	19|3	GT1
Steiner (2017)	cohort	2016.4	OPr ± D	14	14 (100.0)	41.9 ± 3.2	9 (66.7)	NA	NA	11|3
Milazzo (2017)	observational study	2014.12–2015.12	OPrD±R	22	20 (90.9)	51.5 ± 3.1	NA	NA	NA	GT1

*OPrD ombitasvir/ paritaprevir/ritonavir plus dasabuvir, *OPr* ombitasvir/ paritaprevir/ritonavir; *D* dasabuvir, *R* RBV, *GT* genotype, *TN* treatment-naive, *TE* treatment-experienced, *NA* not applicable

### Efficacy outcomes

#### SVR12 rate

**Fig. 2:**
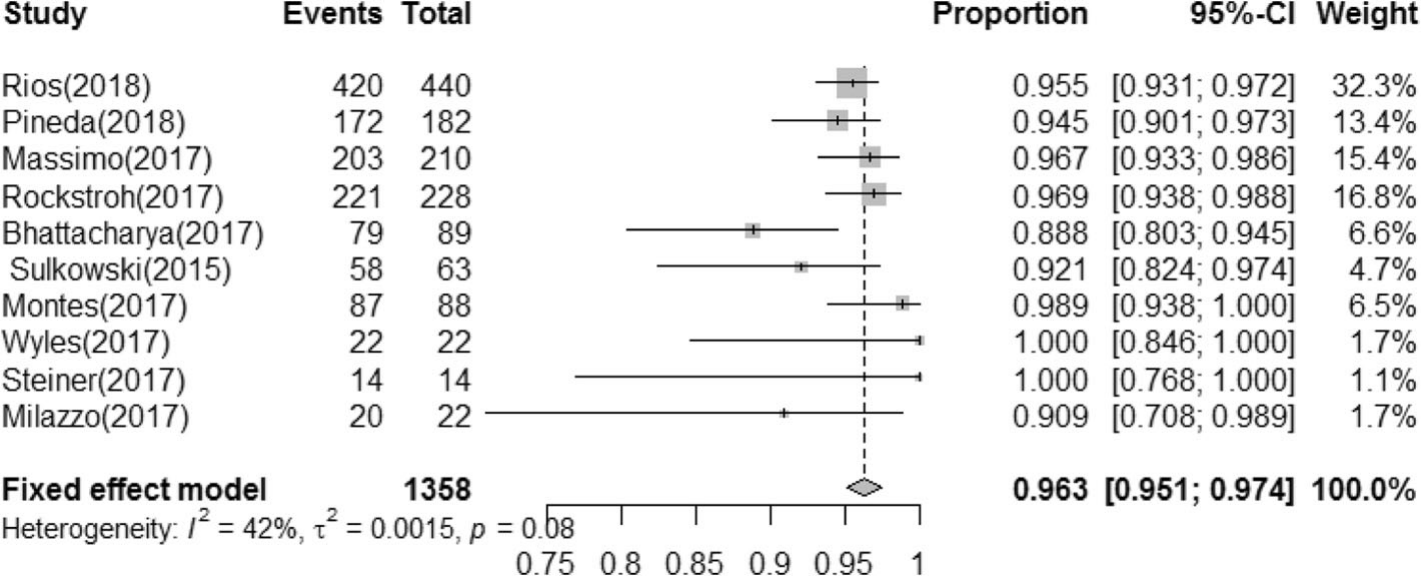
Forest plot of pooled SVR rates of OBV/PTV/r ± DSV ± RBV regimen in patients with HCV/HIV coinfection. *SVR, sustained virologic response; OBV, ombitasvir; PTV, paritaprevir; r, ritonavir; DSV, dasabuvir; RBV, ribavirin; CI, confidence interval

A total of 1296 effective cases in 1358 treated patients were found in the DAA group. The pooled estimated rate of SVR12 was 96.3% (95% CI: 95.1–97.4) (Fig. 2). Based on the different duration of treatment, regimens, genotypes, history of treatment, and the presence or absence of cirrhosis, we subsequently performed subgroup analyses shown in Table 2. Six studies including 470 patients provided data for subgroup analysis by duration of treatment. The 12 weeks treatment subgroup presented the SVR12 rate of 96.2% (394/415), while the SVR12 rate of the 24 weeks treatment subgroup was 96.6% (52/55). Among patients that received the OBV/PTV/r + DSV ± RBV regimen, 96.4% (811/850) achieved SVR; in comparison, 98.9% (152/159) of patients treated with OBV/PTV/r ± RBV achieved SVR. Rate of SVR12 was 96.2% (95% CI, 94.8–97.4) for patients with HCV-GT1 (*n* = 1076), while those with GT4 (*n* = 156) infection had SVR12 rate of 98.8% (95% CI, 95.1–100.0). Five studies including 725 patients provided data for subgroup analysis by treatment history. The SVR12 rates for the TN and TE were 96.8 and 98.9%, respectively. Six studies including 1165 patients provided data for subgroup analysis by presence/absence of liver cirrhosis. SVR rates for those with or without cirrhosis were similar. Pooled SVR rate was 97.8% (95% CI, 94.6–99.8) for patients with cirrhosis (*n* = 230) and 96.7% (95% CI, 95.3–97.8) without cirrhosis (*n* = 935).

**Table 2:** SVR12 by the different duration of treatment, regimens, genotypes, treatment history, and the presence or absence of cirrhosis

Response	SVR12 (*N* = 1358)		Heterogeneity		*P*^b^-value	Studies
	Total, n/N	Rate (95%CI)	*I*^*2*^(%)	*P* ^a^		
Overall	1296/1358	96.3 (95.1–97.4)	42	0.08		10
By the different duration of treatment					0.9237	
12 weeks	394/415	96.2 (92.6–98.8)	41	0.13		6
24 weeks	52/55	96.6 (82.5–100.0)	65	0.09		2
By regimens					0.8563	
OBV/PTV/r ± DSV ± RBV	811/850	96.4 (94.8–97.7)	25	0.21		10
OBV/PTV/r ± RBV	152/159	98.9 (95.3–100.0)	0.0	0.99		3
By genotypes					0.8424	
1	1025/1076	96.2 (94.8–97.4)	38	0.13		8
4	149/156	98.8 (95.1–100.0)	0	0.99		3
By treatment history					0.8281	
TN patients	423/440	96.8 (94.8–98.5)	41	0.15		5
TE patients	273/285	98.9 (96.4–100.0)	50	0.09		5
By the presence or absence of cirrhosis					0.3887	
Cirrhosis	218/230	97.8 (94.6–99.8)	43	0.12		6
Non-cirrhosis	899/935	96.7 (95.3–97.8)	20	0.28		6

*OBV ombitasvir; *PTV* paritaprevir, *r* ritonavir, *DSV* dasabuvir; *RBV* ribavirin, *TN* treatment-naive, *TE* treatment-experienced, *CI* confidence interval

^a^Test of heterogeneity

^b^Test for subgroup differences

#### Subgroup analysis for HCV genotype 1

We conducted subgroup analyses of SVR12 in HCV-GT1 infected patients by different duration of treatment, regimens, treatment history, the presence and absence of cirrhosis, baseline HCV RNA, CD4 cell counts, Platelet counts, and IL 28B genotype in the Table 3. In HCV GT1 infected patients, the 12 weeks treatment subgroup presented SVR12 rate of 96.2% (364/384), while the SVR12 rate of the 24 weeks treatment subgroup was 96.6% (52/55). Among patients that received the OBV/PTV/r + DSV regimen, 97.0% (163/170) achieved SVR; in comparison, 95.8% (350/368) of patients treated with OBV/PTV/r + DSV + RBV achieved SVR. In HCV-GT1 infected patients, the SVR12 rate of the GT1a subgroup was slightly similar to the GT1b subgroup (96.2% vs. 95.9%). Pooled SVR rate was 97.6% (95% CI, 94.2–99.7) for patients with cirrhosis (*n* = 219) and 96.9% (95% CI, 95.5–98.2) without cirrhosis (*n* = 781). Additionally, regarding other sub-analysis for HCV-GT1 patients, there were also no significant differences between the subgroups by prior HCV treatment history (96.9%vs 98.7%, *P* = 0.6859), baseline HCV RNA < 6 log10 IU/m L (97.0% vs 94.0%, *P* = 0.3939), CD4 cell > 500 counts/mm^3^ (96.9% vs 97.8%, *P* = 0.7810), Platelet counts ≤100,000 μL (98.2% vs 97.2%, *P* = 0.7925) and IL 28B CC genotype (97.4% vs 97.9%, *P* = 0.9685).

**Table 3:** Subgroup analysis of SVR12 for GT1

Response	GT1 (N = 1076)		Heterogeneity		*P*^b^-value	Studies
	Total, n/N	Rate (95%CI)	*I*^*2*^(%)	*P* ^a^		
Overall	1025/1076	96.2 (94.8–97.4)	38	0.13		10
By the different duration of treatment					0.9668	
12 weeks	364/384	96.2 (92.5–98.9)	38	0.15		6
24 weeks	52/55	96.6 (82.5–100.0)	65	0.09		2
By regimens					0.8742	
OBV/PTV/r + DSV	163/170	97.0 (93.3–99.5)	0	0.41		4
OBV/PTV/r + DSV + RBV	350/368	95.8 (91.6–98.8)	56	0.06		5
By genotypes					0.6031	
1a	583/608	96.2 (94.5–97.7)	49	0.10		5
1b	311/326	95.9 (93.3–98.0)	14	0.33		5
By treatment history					0.6859	
TN patients	407/423	96.9 (94.9–98.5)	41	0.15		5
TE patients	262/274	98.7 (96.1–100.0)	46	0.12		5
By the presence or absence of cirrhosis					0.2581	
Cirrhosis	207/219	97.6 (94.2–99.7)	45	0.11		6
Non-cirrhosis	753/781	96.9 (95.5–98.2)	20	0.28		6
By baseline HCV RNA					0.3939	
<6 log10 IU/m L	293/304	97.0 (87.8–100.0)	89	<0.01		3
≥6 log10 IU/m L	160/171	94.0 (89.6–97.4)	0	0.87		3
By CD4 cell counts/mm^3^					0.7810	
≤500	129/132	97.8 (94.2–99.8)	0	0.71		2
>500	246/254	96.9 (94.3–98.8)	0	0.38		2
Platelet counts/μL					0.7925	
≤100,000	37/38	98.2 (89.8–100.0)	0	0.41		2
>100,000	338/348	97.2 (95.1–98.7)	0	0.38		2
By IL 28B genotype^c^					0.9685	
CC	37/38	97.4 (89.0–100.0)	–	–		1
Non-CC	101/105	97.9 (93.3–100.0)	71	0.06		2

*OBV ombitasvir, *PTV* paritaprevir, *r* ritonavir, *DSV* dasabuvir, *RBV* ribavirin, *GT* genotype, *TN* treatment-naive, *TE* treatment-experienced, *CI* confidence interval

^a^Test of heterogeneity

^b^Test for subgroup differences

^c^Only one article had the SVR rate of IL 28B CC genotype, thus, there were no test of heterogeneity for it

#### Subgroup analysis for reported HCV treatment regimens

We conduct further analysis of SVR12 for all reported HCV treatment regimens in the Additional file 1: Table S1. Among patients that received the 12-week OBV/PTV/r + RBV regimen, 95.2% (129/136) achieved SVR. The SVR12 rates of 12-week OBV/PTV/r + DSV with or without RBV regimen were 96.6% and 93.3% separately. The 24-week OBV/PTV/r + DSV + RBV regimen showed a SVR rate of 90.6% (29/32). The SVR12 rates of 12-week OBV/PTV/r and 12-week OBV/PTV/r + RBV regimen were both 100%, but the number of the patients were only 3 and 11, respectively.

### Safety

Only three studies have reported the incidence of AEs and SAEs in HCV GT 1/4 infected patients treated with OBV/PTV/r ± DSV ± RBV, and both shown that the treatment regimen was generally well tolerated, with only 2 patients discontinuing due to AE in the 678 coinfected patients, 7 virological breakthrough and 8 virological relapse in the 741 coinfected patients. The two persons who were discontinuing due to an AE: One patient was because of insomnia and the other was a patient in Child-Pugh-Turcotte (CPT) class B, with prior decompensations, who developed hepatic encephalopathy and ended up dying because of liver failure 4 weeks after starting treatment. The pooled estimated AEs and SAEs rate was 73.9% (95%CI: 38.1–97.6) and 2.7% (95% CI: 0.0–9.5) (Table 4). Furthermore, the common AEs were anaemia (34.3%), fatigue (23.9%), diarrhea (14.5%), headache (14.5%), nausea (13.9%), pruritus (10.1%), Insomnia (8.2%), and irritability (2.7%).

**Table 4:** Rate of safety outcomes in HCV/HIV coinfected patients

Outcomes	Safety		Heterogeneity		Studies
	Total, n/N	Rate%(95%CI)	*I*^*2*^(%)	*P*	
Virological breakthrough	7/741	0.2 (0.0–1.0)	0	0.78	7
Virologica relapse	8/741	0.3 (0.0–1.2)	0	0.72	7
Discontinuation due to AE	2/678	0.0 (0.0–0.1)	0	0.52	6
AEs	301/460	73.9 (38.1–97.6)	98	< 0.01	3
SAEs	16/460	2.7 (0.0–9.5)	85	< 0.01	3

*OBV ombitasvir, *PTV* paritaprevir, *r* ritonavir, *DSV* dasabuvir, *RBV* ribavirin, *CI* confidence interval, *AEs* adverse events, *SAEs* serious adverse events

### Publication bias

**Fig. 3:**
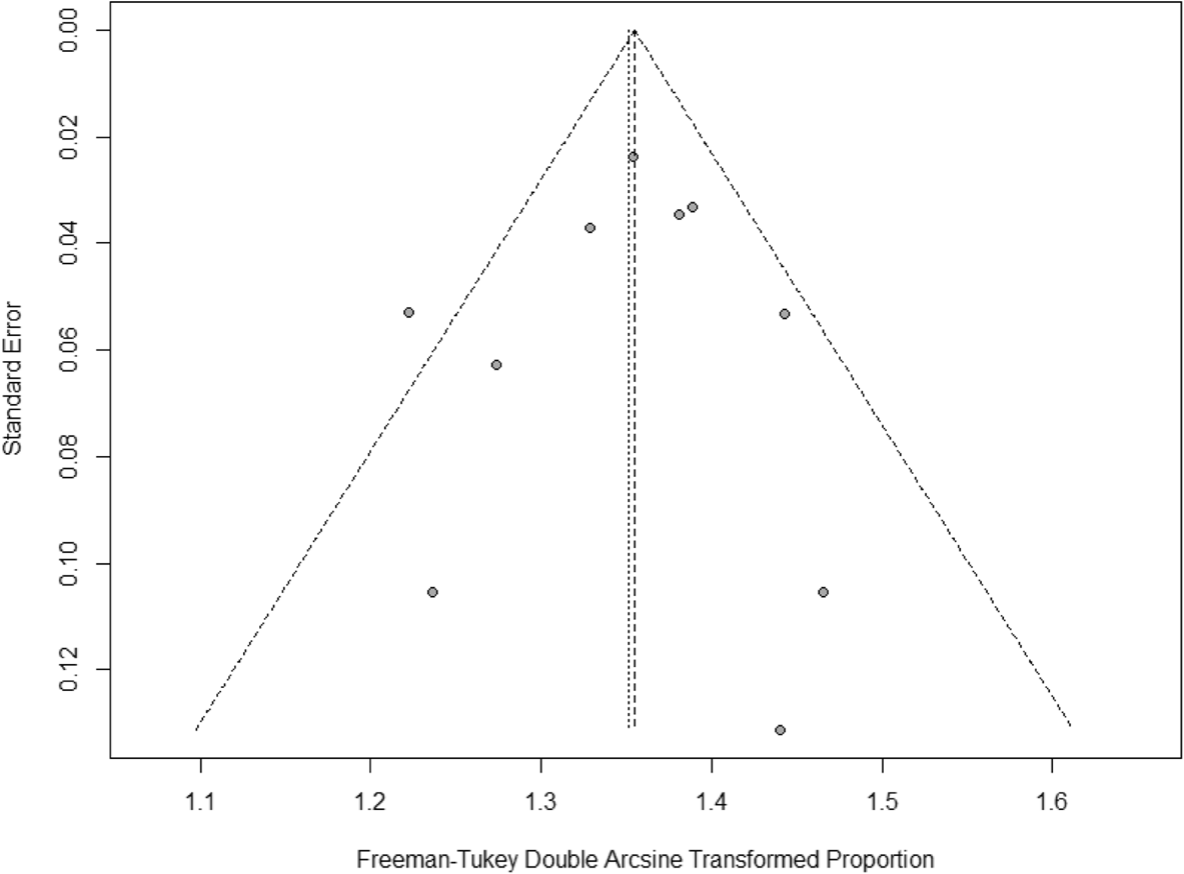
Funnel plot for the evaluation of publication bias (a)

**Fig. 4:**
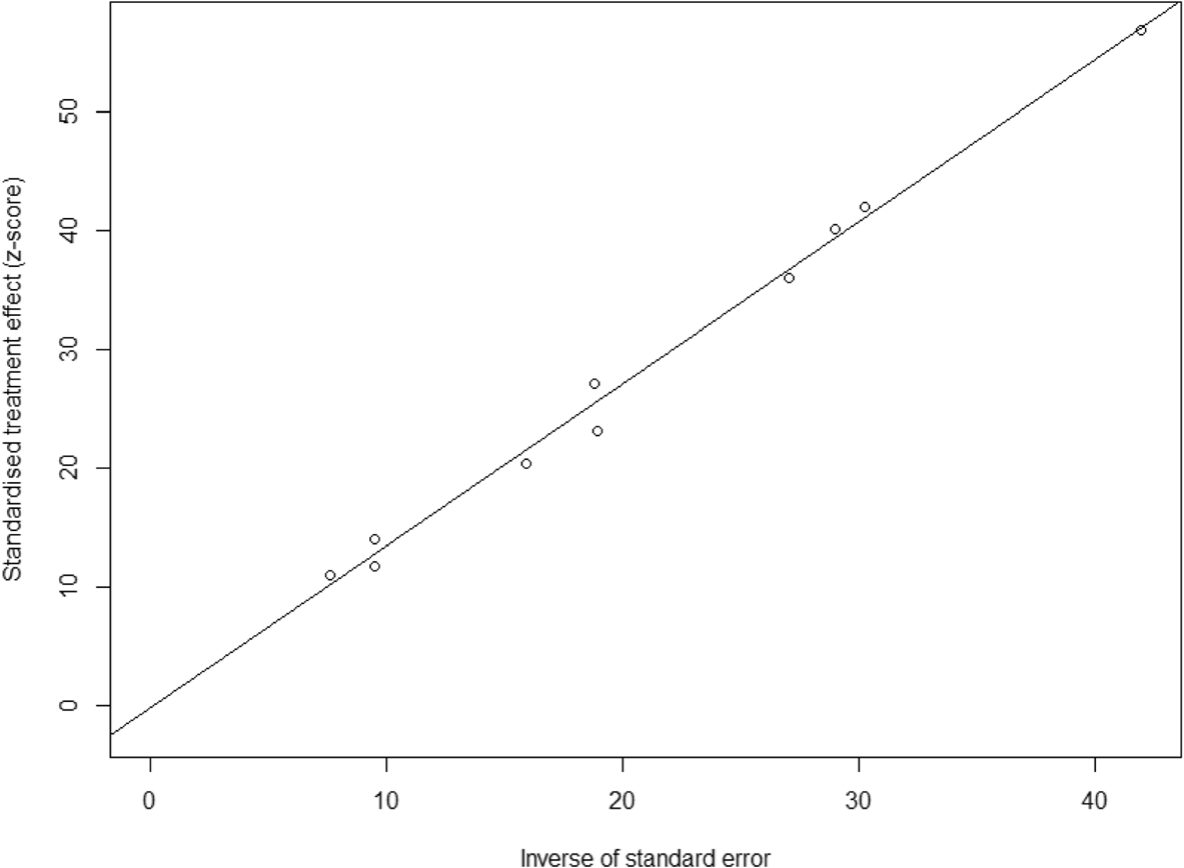
Egger’s funnel plot for the evaluation of publication bias (b)

The funnel plot for the SVR12 rate was shown in Fig. 3 and 4. The studies were distributed closely within the 95% confidence interval axis, indicating no obvious publication bias. In addition, the Egger’s test for evaluating publication bias also showed no statistical significance (*t* = − 0.257 *P* = 0.804).

## Discussion

The post hoc analysis showed a high efficacy for OBV/PTV/r ± DSV ± RBV regimen in treatment of HIV/HCV coinfection especially, regardless of the duration of treatment, regimens, HCV genotypes, history of treatment, and the presence or absence of cirrhosis. The overall pooled SVR rates ranged from 96.2% to 98.9% for the different subgroup. The results were consistent with a recent study by Juan Berenguer, which showed that IFN-free DAA regimens were highly effective in coinfected patients [6].

Current guidelines recommend that HIV/HCV-coinfected persons could be treated with the approach followed for non-HIV-infected individuals because the efficacy of currently licensed DAA regimens in coinfected individuals did not appear to be lower than HCV-monoinfected patients [2, 6]. Heiner Wedemeyer’s meta-analysis of real-world data for OBV/PTV/r ± DSV ± RBV treatment of HCV-monoinfected patients found that the overall SVR12 rates were 96.8% (95% CI 95.8–97.7) for GT1(*n* = 5046) and 98.9% (95% CI 94.2–100) for GT4(*n* = 112) [25]. In our study, the overall SVR12 rates were 96.2% (95% CI 94.8–97.4) for GT1 (*n* = 1076) and 98.8% (95% CI 95.1–100.0) for GT4 (*n* = 156) in HIV/HCV coinfected subjects. Recent studies also found the rates of SVR12 of HCV/HIV-infected patients treated with DAAs were similar to those of HCV-monoinfected patients under real-life conditions [24]. Additionally, previous research have found that the pooled efficacy of peg-IFN and RBV in the treatment of HCV and HIV-coinfected patients was 33.3%(95% CI 27.3–44.2, *n* = 748) [26], which was obviously lower than our pooled SVR rate of 96.4% (95%CI: 94.8–97.7) for patients received OBV/PTV/r ± DSV ± RBV combined regimen. Therefore, we could reason that the OBV/PTV/r ± DSV ± RBV treatment of patients with HIV/HCV GT1 or 4 infection from over 1300 patients demonstrated high effectiveness.

Lower SVR rates may be expected in cirrhotic patients and particularly TE cirrhotic patients treated in routine clinical practice [13, 20]. However, our subgroup analysis of SVR12 rates for whether cirrhotic or non-cirrhotic, TN or TE patients were highly effective which were all over 96%. This is consistent with other people’s research for HIV and HCV coinfection which showed that neither cirrhosis nor prior HCV TE had a statistically significant impact on SVR rates for people treated with this regimen [17, 21]. Cirrhotic in HCV-monoinfected patients also had very high rates of SVR12, similar rate to noncirrhotic. SVR rates for TE and TN patients in the HCV-monoinfected population were over 90.9% [27–29]. Thus, we can conclude that OBV/PTV/r ± DSV ± RBV regimen was generally highly effective in cirrhosis or TE patients.

Genotype 1 HCV is the most prevalent type all around the world (accounting for ~ 50% of all HCV infections) and is also the most difficult type to cure [30, 31]. Correspondingly, 1076 patients in the study were infected with HCV GT 1a or 1b, which were the predominant genotypes. The SVR12 rates were high in OBV/PTV/r ± DSV ± RBV for HIV/HCV GT1 coinfected patients regardless of the duration of treatment, regimens, HCV genotypes, history of treatment, and the presence or absence of cirrhosis, baseline HCV RNA, CD4 cell counts, Platelet counts, and IL 28B genotype. As for the regimens, RBV was added to IFN free regimen only in “difficult to treat” patients, including those with GT1a and cirrhotic GT1b individuals. However, Hussien Ahmed’s systematic review showed that whether adding RBV to an OBV/PTV/r and DSV regimen in the treatment for HCV genotype 1, it observed no difference in the sustained virological response [32]. We also found high pooled SVR rates both in with and without RBV regimen (95.8% vs. 99.8%). RBV did not seem to have an impact on the achievement of SVR. Futhermore, SVR rates for different regimen of genotype 1a and genotype 1b separately were shown in Additional file 1: Table S2. Although, the sample size of other regimen for HCV genotype 1a and genotype 1b were not enough, an OBV/PTV/r + DSV + RBV regimen in the treatment for HCV genotype 1a and OBV/PTV/r + DSV regimen in the treatment for HCV genotype 1b all achieve high efficacy. Therefore, future guidelines should consider the possibility of removing RBV from this combination regimen.

Treatment of HCV-HIV coinfected patients with the OBV/PTV/r regimen resulted in low virological breakthrough and relapse. This study suggested that OBV/PTV/r ± DSV ± RBV did not increase the possibility of discontinuation, virological breakthrough and virological relapse. Our study also found that patients receiving this combination regimen had a low occurrence rate of SAEs and AEs. There were few treatment discontinuations due to AEs, and the overall safety profile was consistent with those receiving OBV/PTV/r + DSV + RBV [12, 13, 33]. In Gonzales’s retrospective review, patients who developed AE were more often Caucasian and were more frequently treated with OBV/PTV/r + DSV + RBV [34]. Previous studies showed that RBV is responsible for a considerable number of side effects, highlighting anemia and rash, especially compared with second-generation DAAs [35]. Nevertheless, there was no sufficient evidence to prove the safety outcome, due to the few coinfected patients included in this study. Therefore, more patients should be included in studies to obtain sufficient evidence.

Our study has several limitations. First, most of the studies included were uncontrolled trials due to the special management of HIV patients. Eligible studies included three clinical trials, four observational studies and three cohort studies. Thus, it limited the ability to derive definitive conclusions regarding the safety and efficacy of this regimen. Second, Although the OBV/PTV/r ± DSV ± RBV regimens was recommended for the GT4 patients, HCV with GT4 was relatively uncommon and there were insufficient data for a meaningful pooled estimate for SVR rate for HCV GT4. We did not show a subgroup analysis for HCV GT4. Therefore, large scale of patients and robust results are needed for SVR rate of HCV GT4. Moreover, Limited articles were included and some important data have not been reported in the articles we included, a complete comparison for 8 different regimens (O/P/r only, O/P/r + D, O/P/r + RBV, and O/P/r + D + RBV; each either administered for 12 weeks or 24 weeks) was lacked. Considering above problems, further studies are still required in the near future.

## Conclusion

The current comprehensive analysis showed a high efficacy for the OBV/PTV/r ± DSV ± RBV regimen in the treatment of HCV-HIV coinfected patients, regardless of genotypes, history of treatment and the presence or absence of cirrhosis. It is important to confirm the effectiveness of new HCV therapies in real-world settings, as well as to provide better therapeutic alternatives to patients [25]. Future studies with a larger sample size are required to investigate the efficacy of this regimen and to establish the evidence about the safety outcomes.

## Additional file

Additional file 1: **Table S1**. Details of SVR12 for all reported HCV treatment regimens. **Table S2**. Treatment outcomes for HCV genotype 1a and genotype 1b with or without RBV. (DOCX 18 kb)

## Abbreviations

2-DAA: Two direct-acting antivirals
AEs: Any adverse events
CI: Confidence intervals
DAAs: Direct-acting antiviral agents
DSV: Dasabuvir
GT: Genotype
HCC: Hepatocellular carcinoma
HCV: Hepatitis C virus
HIV: Human immunodeficiency virus
OBV: Ombitasvir
peg IFN: Pegylated interferon
PTV: Paritaprevir
r: Ritonavir
RBV: Ribavirin
SAEs: Serious adverse events
SVR: Sustained virological response
SVR12: Sustained virological response after 12 weeks
TE: Treatment-experienced
TN: Treatment-naïve
