# Supplementary material for: Effectiveness of ombitasvir/paritaprevir/ritonavir, dasabuvir for HCV in HIV/HCV coinfected subjects: a comprehensive analysis
Source: Virol J. 2019 Jan 17;16:11. doi: 10.1186/s12985-018-1114-4 (PMC6337763; doi:10.1186/s12985-018-1114-4)
Supplement: Supplementary file 1 — Table S1. Details of SVR12 for all reported HCV treatment regimens. Table S2. Treatment outcomes for HCV genotype 1a and genotype 1b with or without RBV. (DOCX 18 kb) [file 12985_2018_1114_MOESM1_ESM.docx]

Table S1. Details of SVR12 for all reported HCV treatment regimens.

| Response | SVR12 | | Heterogeneity | | Studies | GT |
| --- | --- | --- | --- | --- | --- | --- |
|  | Total, n/N | Rate (95%CI) | *I^2^* (%) | *P* |  |  |
| OBV/PTV/r (12wks) | 3/3 | 100.0(29.2-100.0) | -- | -- | 1 | GT4 |
| OBV/PTV/r + RBV (12wks) | 129/136 | 95.2(90.6-98.5) | 0 | 0.85 | 2 | GT4 |
| OBV/PTV/r+ RBV (24wks) | 11/11 | 100.0(71.5-100.0) | -- | -- | 1 | GT4 |
| OBV/PTV/r + DSV (12wks) | 78/82 | 96.6(90.6-99.9) | 23 | 0.27 | 3 | GT1b |
| OBV/PTV/r + DSV + RBV (12wks) | 106/115 | 93.3(87.6-97.6) | 51 | 0.13 | 3 | GT1 |
| OBV/PTV/r + DSV + RBV (24wks) | 29/32 | 90.6(75.0-98.0) | -- | -- | 1 | GT1 |

* OBV, ombitasvir; PTV, paritaprevir; r, ritonavir; DSV, dasabuvir; RBV, ribavirin; GT,genotype ; CI, confidence interval.

Table S2. Treatment outcomes for HCV genotype 1a and genotype 1b with or without RBV.

| Response | SVR | | Heterogeneity | | Studies |
| --- | --- | --- | --- | --- | --- |
|  | Total, n/N | Rate (95%CI) | *I^2^* (%) | *P* |  |
| **GT1a** |  |  |  |  |  |
| OBV/PTV/r + DSV | 8/8 | 100.0(79.6-100.0) | -- | -- | 1 |
| OBV/PTV/r + DSV + RBV | 415/433 | 95.8(91.8-98.5) | 62 | 0.05 |  |
| **GT1b** |  |  |  |  |  |
| OBV/PTV/r + DSV | 142/137 | 99.8(97.0-100.0) | 22 | 0.28 | 4 |
| OBV/PTV/r + DSV + RBV | 8/8 | 100.0(79.6-100.0) | -- | -- | 1 |

* OBV, ombitasvir; PTV, paritaprevir; r, ritonavir; DSV, dasabuvir; RBV, ribavirin; GT,genotype ; CI, confidence interval.
